# Clinical advisors at NHS 111 improve accuracy for paediatric patients and their advice is more reliably followed: a retrospective observational cohort study

**DOI:** 10.1136/archdischild-2025-328896

**Published:** 2025-10-22

**Authors:** Jen Lewis, Rebecca M Simpson, Tony Stone, Nicola Ennis, Nicola Jay, Susan Croft, Richard Pilbery, Suzanne M Mason

**Affiliations:** 1 SCHARR, Division of Population Health, The University of Sheffield, Sheffield, England, UK; 2 Sheffield Children’s Hospital NHS Foundation Trust, Sheffield, England, UK; 3 Nottingham and Nottinghamshire ICS, Nottingham, England, UK; 4 Sheffield Teaching Hospitals NHS Foundation Trust, Sheffield, UK; 5 Yorkshire Ambulance Service NHS Trust, Wakefield, UK; 6 Data Connect, The University of Sheffield, Sheffield, UK

**Keywords:** Emergency Care, Paediatric Emergency Medicine, Health services research, Child Health Services

## Abstract

**Objective:**

To determine whether National Health Service (NHS) 111 advice regarding paediatric patients given by clinically trained health advisors (CHAs) is, as previously found for adult patients, less risk-averse, more accurate and complied with more than that given by non-clinically trained health advisors (NHAs)

**Design:**

Retrospective observational study using routinely collected, linked NHS urgent care data.

**Setting:**

NHS 111 triaging services in Yorkshire and the Humber, 2014–2017.

**Patients:**

Children (<16 years) who were the subject of a call to NHS 111.

**Main outcome measures:**

The recommendation given, whether the patient attended the emergency department (ED) within 48 hours and if so whether the patient was admitted to hospital, or considered ‘non-urgent’. Adjusted logistic regressions were used for analysis.

**Results:**

972 221 calls were analysed (26.5% CHA; 73.5% NHA). CHAs were more likely than NHAs to recommend guardian/self-care (OR 45, 95% CI 44 to 46), and less likely to recommend ambulance dispatch (OR 0.5; 95% CI 0.48 to 0.51), ED attendance (OR 0.79; 95% CI 0.77 to 0.8) or primary care (OR 0.163; 95% CI 0.161 to 0.165). Patients were less likely to attend ED following guardian/self-care recommendations from CHAs versus NHAs (OR=0.64; 95% CI 0.56 to 0.74), but no more likely to be admitted if they did attend (OR 1.2; 95% CI 0.8 to 1.8). Callers were more likely to terminate a call before receiving a formal recommendation from a CHA (OR 2.02; 95% CI 2.0 to 2.1). Call-terminators were less likely to attend ED (OR 0.128; 95% CI 0.12 to 0.13) and more likely to be considered non-urgent if attending ED (OR 1.23; 95% CI 1.2 to 1.3) if advised by a CHA.

**Conclusions:**

Paediatric patient journeys suggest triage by CHAs is less risk-averse and more accurate. Patients are more likely to avoid attending ED if advised to by a CHA. Callers who terminate a call early may typically represent the ‘worried well’. CHAs may better identify these patients and discourage them from attending ED in prerecommendation conversation. This has implications for the cost-benefit balance of NHS 111 staffing.

**WHAT IS ALREADY KNOWN ON THIS TOPIC**

**WHAT THIS STUDY ADDS**

- Clinically trained health advisors (CHAs) were 45 times as likely to recommend staying home as non-clinically trained health advisors (NHAs). Patients were around 36% less likely to attend the emergency department (ED) if advised to stay home by a CHA versus NHA. Patient outcomes at ED suggest CHAs give more accurate recommendations, particularly for low-acuity patients. CHAs may be better at identifying the ‘worried well’ and discouraging them from unnecessary UEC use even if the caller terminates before a formal recommendation.

**HOW THIS STUDY MIGHT AFFECT RESEARCH, PRACTICE OR POLICY**

- NHS 111 has the potential to safely reduce paediatric demand at UEC with a greater proportion of CHAs. A cost-benefit analysis would be useful to understand the balance of improvements at UEC services against availability and cost of staffing NHS 111.

## Introduction

 National Health Service (NHS) 111 is a free-to-use 24/7 telephone triage and advice service available throughout the UK, aiming to direct patients with urgent but non-life-threatening health concerns to the appropriate level of care. In turn, it aims to reduce pressure on urgent and emergency care (UEC) services, including emergency departments (EDs). The service is staffed mainly by ‘Non-clinically trained Health Advisors’ (NHAs), who use a digital clinical decision support system known as NHS Pathways,^1^ which is designed to support decision-making regarding the most appropriate recommendation for the caller. In the most urgent cases, the call handler may contact an ambulance service on behalf of the caller. In the least urgent cases, the caller may be given advice and recommended to stay home and care for themselves (or be cared for by a parent/guardian). Calls are typically answered by an NHA who performs provisional triaging following NHS Pathways. After triage, a final recommendation is given for the appropriate level of care. In complex cases or where a clinical opinion is required, NHAs may seek support from clinically trained health advisors (CHAs). A CHA may speak with the patient via direct transfer or a subsequent callback. CHAs during the time of this study were mainly paramedics with skills in handling prehospital emergencies, and nurses with a range of backgrounds. CHAs have the flexibility to integrate clinical knowledge with the guidance provided by NHS Pathways, allowing them to diverge from standard algorithms and give a more appropriate recommendation.

Despite its aims, evidence suggests NHS 111 may actually *increase* UEC use.^2 3^ This may result from the lower level of clinical knowledge of the NHAs comprising most of the advisory staff, or possible risk aversion of NHAs or the NHS Pathways algorithm. Previously, we found that for adult callers, triaging and its accuracy were associated with the advisor’s clinical skill^4^: NHAs were more likely to direct low-acuity callers to ED or dispatch an ambulance than CHAs. We also identified lower patient compliance with advice given by an NHA.

Evidence suggests adults use UEC differently for themselves and their children; for example, a higher proportion of paediatric ED attendances are non-urgent^5^ (21.4%) than adult attendances^6^ (15.1%). Reasons for this may include parental anxiety, difficulty judging the urgency of children’s acute illnesses and the need for reassurance.^7 8^ Although resources exist to support parental decision-making for acutely unwell children, these may not prevent parents seeking UEC unnecessarily.^9^ A Department of health report revealed that some parents perceived NHAs as unskilled and were distrustful of NHS Pathways.^10^ One study suggests clinical input at NHS 111 is associated with lower ED attendance rates for children.^11^ However, it is unclear whether clinical NHS 111 triaging for children is more accurate, nor whether parental trust translates into better compliance with CHA advice.

We aimed to compare triaging outcomes and paediatric patient behaviour for CHAs versus NHAs. Following previous findings for adults, we hypothesise that CHAs triage more conservatively than NHAs, that their advice is more accurate and that patients or their guardians are more likely to follow CHA advice.

## Methods

### Data sources

This study used routinely collected, record-level NHS data held in the University of Sheffield Centre for Urgent and Emergency Care research database (CUREd). This comprises linked data regarding ED attendances, acute hospital admissions, ambulance callouts and NHS 111 calls from Yorkshire and the Humber. The construction and administration of the CUREd database are detailed elsewhere.^12^

We used a CUREd extract comprising deidentified data from all NHS 111 calls made regarding patients under 16 between 1 February 2014 and 31 March 2017. This start date was chosen as the time when NHS 111 was fully implemented.^3^ Calls were linked to any attendance at a type 1 ED made within 48 hours of the NHS 111 call. A type 1 ED is a consultant-led 24-hour service with full resuscitation facilities and designated accommodation for the reception of ED patients. Calls were also linked with any acute hospital admission within 7 days of the call, indicated by an admitted patient care record for that patient.

### Cohorts

We defined two cohorts. The NHA cohort included calls handled exclusively by an NHA. The CHA cohort included calls that involved input from a CHA, which could be during the initial call or a subsequent callback. While some callers may have spoken exclusively with a CHA, this likely represents a negligible fraction of calls. Therefore, the CHA cohort is assumed to have spoken initially with an NHA.

### Exclusions

We excluded cases where the final recommendation was missing, the caller was referred without triage or the final recommendation was to speak with a CHA from the service. These were likely cases where the caller expected to speak with a CHA, but a callback was never received or not recorded.

### Outcomes

**Figure 1:**
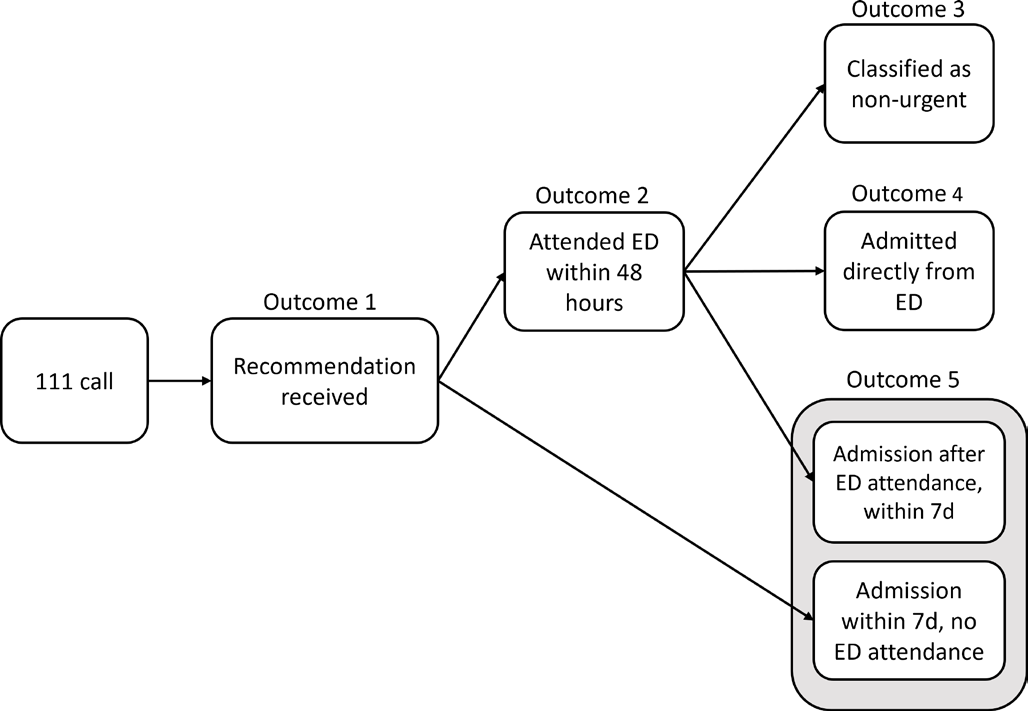
Flow of patient outcomes following an NHS 111 call. ED, emergency department; NHS, National Health Service.

There were five main outcomes (figure 1):

1. Type of recommendation to the caller (guardian/self-care; primary care; attend ED; ambulance dispatch; or other);
2. Whether the patient made an ED attendance within 48 hours;
3. For those attending ED, whether the attendance was classified as non-urgent;
4. For those attending ED, whether the patient experienced an acute admission directly from ED;
5. Whether the patient experienced an acute admission within 7 days, where the admission was not linked to an ED attendance within 48 hours of the call. This may be because they had not attended ED, they had initially been discharged or referred elsewhere, or had attended ED later than 48 hours after the call. This indicated cases of general deterioration after the initial call.

### Statistical analysis

We summarised demographic variables for the subjects of each call, the main presenting problem (after categorisation into five categories as described in online supplemental table 1) and each outcome, overall and by cohort.

We carried out logistic regressions to quantify the impact of the advisor on each outcome. Outcomes were analysed separately for each recommendation to avoid complex interactions and ease interpretation. The main predictor was the advisor type (reference level was NHA). Covariates were included to adjust for age, gender, Index of Multiple Deprivation quintile, the category of presenting problem and whether the call was in-hours or out-of-hours. In-hours is considered 08:00–18:00 Monday–Friday.^5^ Missing covariates were classified as ‘unknown’ to maximise the sample size.

Analyses were performed in R V.4.3.1. Results are reported to two significant figures, unless more were required to convey meaningful precision.

### Sensitivity analyses

1. As including missing variables as ‘unknown’ can introduce bias, we performed a complete case analysis to assess robustness of the results.
2. For outcome 3, 10.7% of cases could not be definitively calculated due to incomplete or ambiguous data. For sensitivity analysis, we manually imputed these values based on available data.
3. Some call records contained features that were inconsistent with the recorded skill level of the advisor. We performed a sensitivity analysis that excluded or reclassified these calls.

## Results

**Figure 2:**
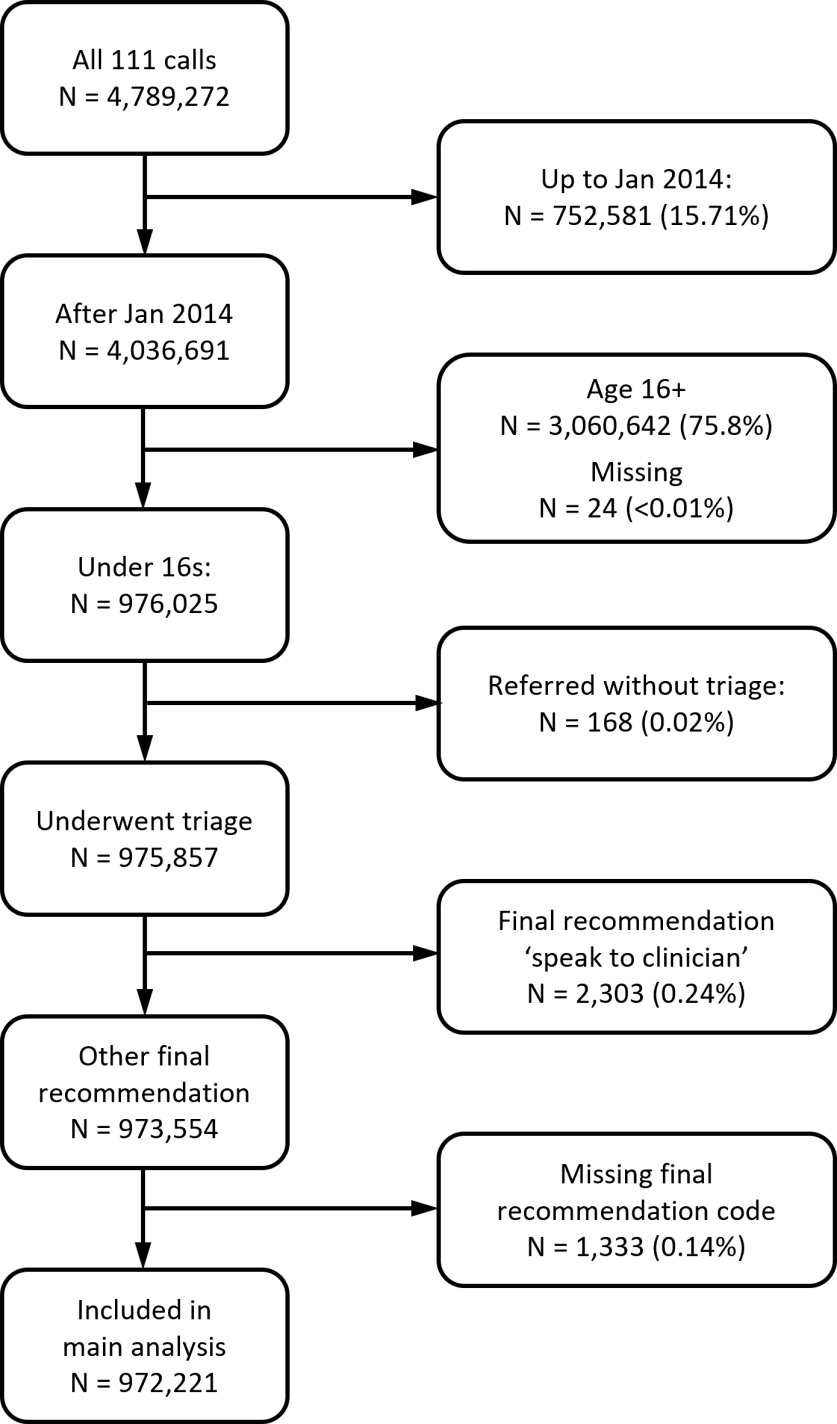
Flowchart illustrating call exclusions from the full CUREd NHS 111 data set and size of final cohort. CUREd, Centre for Urgent and Emergency Care research database; NHS, National Health Service.

Figure 2 shows the cohort identification process. After age and date exclusions, 168 calls were referred without triage (<0.1%) and 2303 calls (0.24%) were excluded based on a recommendation to speak to a CHA but without evidence this occurred. 1333 calls (0.14%) were missing recommendations and were excluded.

There were 972 221 eligible NHS 111 calls between 1 February 2014 and 31 March 2017. 192 330 (19.8%) patients attended ED within 48 hours of the call. Of those, 45 185 (23.5%; 4.6% of calls) were non-urgent and 42 204 (21.9%; 4.3% of calls) were admitted directly to hospital. 28 436 (2.9% of calls) were admitted to hospital within 7 days, but not via a 48-hour ED attendance. 257 486 calls (26.5%) were advised by a CHA and 714 735 (73.5%) by an NHA only.

The data showed significant usage of a final recommendation code labelled ‘Not recommended to attend other service’. Inspection revealed that this identified calls where a caller began or even completed triage but terminated the call before receiving a final recommendation. We analysed this group separately to explore any influence of pre-recommendation discussion or triaging on subsequent patient behaviour.

**Figure 3:**
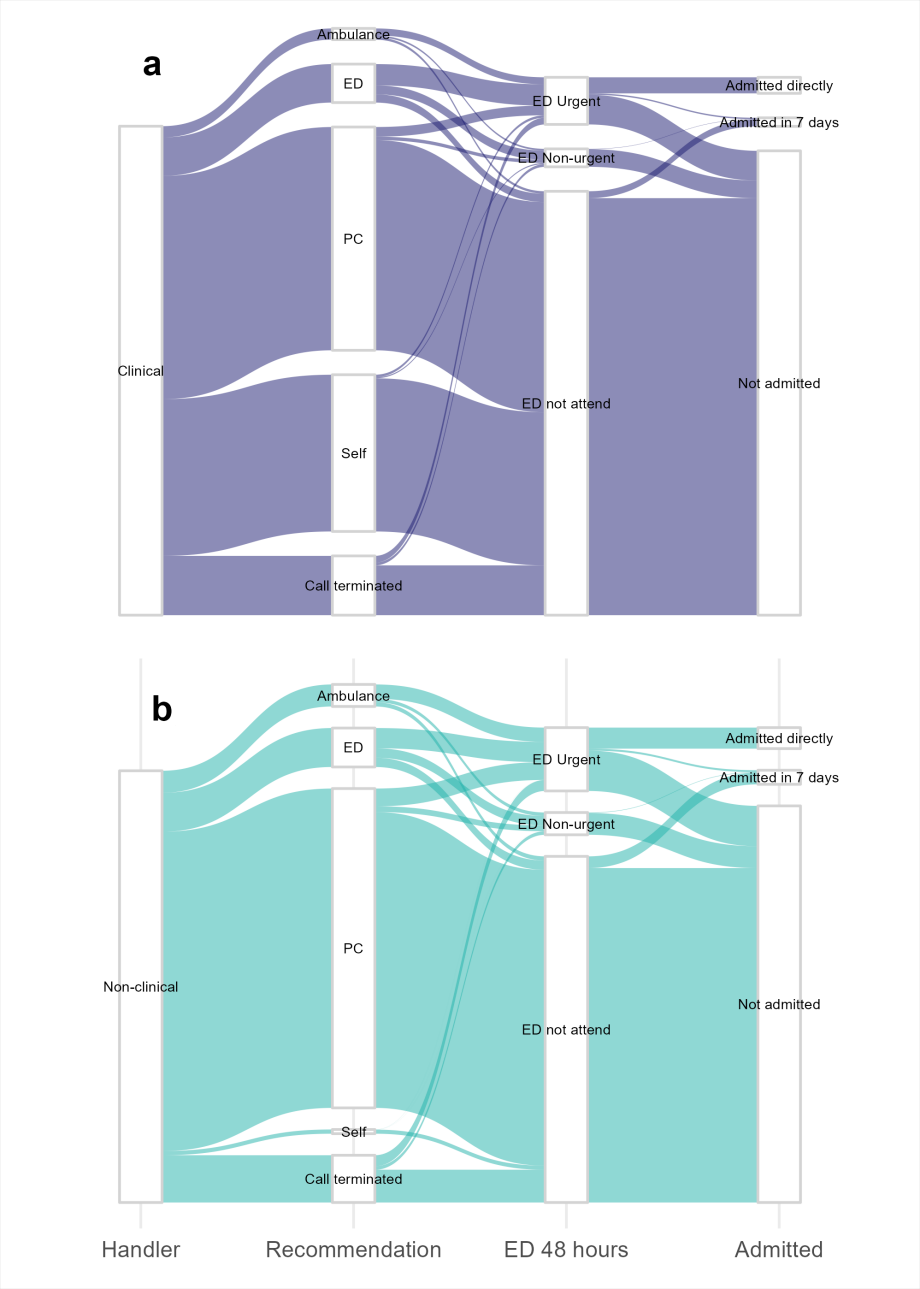
Main patient journeys by volume after an NHS 111 call. (a) Journeys for patients triaged by a CHA. (b) Journeys for patients triaged by an NHA. Pathways including a recommendation of ‘other service’ or where ED urgency level was missing were excluded for visibility (3.2% of data). CHA, clinically trained health advisor; ED, emergency department; NHA, non-clinically trained health advisor; NHS, National Health Service; PC, primary care.

Table 1 summarises demographics and outcomes by cohort (online supplemental table 2 displays these by recommendation). There were no notable demographic differences in patients advised by CHAs and NHAs, though CHA calls tended to have fewer missing data. CHAs advised on slightly more calls with symptom categories of ‘Mixed/unclear’ (58% vs 54%) or ‘Other’ (6.2% vs 3.4%), implying they handle more calls with complex presenting problems. Table 1 shows that more callers advised by CHAs were recommended guardian/self-care than those handled by NHAs alone (31% vs 0.9%). NHAs more frequently recommended primary care (72.5% vs 44.7%), ED attendance (9.7% vs 8.4%) and ambulance dispatch (5.3% vs 2.4%). CHAs recommended ‘other’ services more often (1.3% vs 0.6%), including social services, opticians and midwives, among others. Unadjusted ORs and 95% CIs quantifying crude differences are in online supplemental table 3). Patient journeys by volume are illustrated in figure 3.

**Table 1:** Characteristics and outcomes for all index NHS 111 calls overall and by advisor type

	Advised by CHA Total=257 486	Advised by NHA only Total=714 735	Overall Total=972 221
	Median (IQR)	Median (IQR)	Median (IQR)
Age	2 (1–6)	2 (0–6)	2 (1–6)
	**N (%)**	**N (%)**	**N (%)**
Gender			
Male	134 137 (52)	374 539 (52)	508 676 (52)
Female	123 253 (48)	336 115 (47)	459 368 (47)
Not stated/ Indeterminate	96 (0.04)	4081 (0.6)	4177 (0.4)
IMD Quintile			
1 (most deprived)	98 614 (38)	279 169 (39)	377 783 (39)
2	50 522 (20)	134 140 (19)	184 662 (19)
3	39 439 (15)	101 496 (14)	140 935 (15)
4	39 282 (15)	98 537 (14)	137 819 (14)
5 (least deprived)	28 321 (11)	69 087 (10)	97 408 (10)
None recorded	1308 (0.5)	32 306 (4.5)	33 614 (3.5)
Complaint category			
Non-urgent	59 815 (23)	171 331 (24)	231 146 (24)
Urgent	6759 (2.6)	36 126 (5.1)	42 885 (4.4)
Injury	23 299 (9.0)	65 898 (9.2)	89 197 (9.2)
Mixed/unclear	149 969 (58)	385 745 (54)	535 714 (55)
Other	15 872 (6.2)	24 342 (3.4)	40 214 (4.1)
None recorded	1772 (0.7)	31 293 (4.4)	33 065 (3.4)
Time of call			
In-hours	56 958 (22)	145 796 (20)	202 754 (21)
Out-of-hours	200 528 (78)	568 939 (80)	769 467 (79)
Recommendation			
Self-care	80 395 (31)	6774 (0.9)	87 169 (9.0)
Primary care	115 089 (45)	517 841 (73)	632 930 (65)
Attend ED	21 563 (8.4)	68 989 (9.7)	90 552 (9.3)
Ambulance	6219 (2.4)	38 233 (5.3)	44 452 (4.6)
Other	3280 (1.3)	4230 (0.6)	7510 (0.8)
Call terminated	30 940 (12)	78 668 (11)	109 608 (11)
Attended ED within 48 hours	37 712 (15)	154 618 (22)	192 330 (20)
ED urgency (% of attendances)			
Non-urgent	9292 (25)	35 893 (23)	45 185 (24)
Urgent	24 292 (64)	102 183 (66)	126 475 (66)
Not calculable	4128 (11)	16 542 (11)	20 670 (11)
Admitted from ED (% of attendances)	8270 (22)	33 934 (22)	42 204 (22)
Otherwise admitted in 7 days (% of calls not admitted from ED)	4642 (1.8)	23 794 (3.3)	28 436 (2.9)

CHA, clinically trained health advisor; ED, emergency department; IMD, Index of Multiple Deprivation; NHA, non-clinically trained health advisor; NHS, National Health Service.

Table 2 shows adjusted ORs and 95% CIs. After adjustment, CHAs were 45 times as likely to recommend guardian/self-care as NHAs (OR 45; 95% CI 44 to 46; online supplemental figure S4.1). CHAs were around 84% less likely to recommend primary care (OR 0.163; 95% CI 0.161 to 0.165). CHAs were also less likely to recommend the patient attend ED (OR 0.79; 95% CI 0.77 to 0.8), or to dispatch an ambulance (OR 0.5; 95% CI 0.48 to 0.51). Callers were more likely to terminate the call if advised by a CHA (OR 2.02; 95% CI 2 to 2.1).

**Table 2:** ORs, 95% CIs and N calls analysed for each logistic regression. Reference level was NHA

	Guardian/self-care	Primary care	Attend ED	Ambulance dispatch	Other	Call terminated
Received recommendation	45 (44 to 46)	0.163 (0.161 to 0.165)	0.79 (0.77 to 0.80)	0.50 (0.48 to 0.51)	0.77 (0.73 to 0.82)	2.02 (2.0 to 2.1)
Attended ED within 48 hours	0.64 (0.56 to 0.74) n=87 169	0.78 (0.76 to 0.80) n=632 930	1.0 (0.9 to 1.1) n=90 552	0.9 (0.85 to 0.98) n=44 452	0.8 (0.7 to 0.9) n=7510	0.128 (0.12 to 0.13) n=109 608
Classified as non-urgent*	0.9 (0.7 to 1.2) n=2071	1.07 (1.01 to 1.1) n=44 465	0.97 (0.93 to 1.02) n=63 175	1.0 (0.9 to 1.1) n=33 074	1.7 (1.2 to 2.4) n=789	1.23 (1.2 to 1.3) n=28 086
Admitted from ED	1.2 (0.8 to 1.8) n=2325	0.89 (0.8 to 0.9) n=49 828	1.2 (1.17 to 1.30) n=71 492	0.95 (0.9 to 1.0) n=36 373	0.64 (0.4 to 0.9) n=880	0.78 (0.7 to 0.8) n=31 432
Otherwise admitted in 7 days†	0.7 (0.5 to 0.96) n=87 169	0.68 (0.65 to 0.71) n=632 930	0.94 (0.8 to 1.1) n=90 552	1.0 (0.9 to 1.2) n=44 452	0.33 (0.2 to 0.5) n=7510	0.45 (0.4 to 0.5) 109 608

* This outcome could not be definitively calculated for 20 670 (10.7%) ED attendances. These were excluded from this analysis. These represented a comparable per cent of calls for NHAs (10.7%) and CHAs (10.9%).

† Sex was excluded as a covariate for this outcome as the fully adjusted analyses included very small sample sizes for permutations of covariates including the ‘Indeterminate/Not stated’ sex category resulting in inestimable effect sizes (see online supplemental table 2).

CHAs, clinically trained health advisors; ED, emergency department; NHAs, non-clinically trained health advisors.

Patients were less likely to attend ED within 48 hours if advised by a CHA following all recommendations except when the patient was specifically recommended to attend ED (online supplemental figure S4.2).

Patients who were recommended primary care but who attended ED regardless were more likely to be classified as non-urgent if they were advised by a CHA (OR 1.07; 95% CI 1.01 to 1.1; online supplemental figure S4.3). This was also true for patients recommended to attend other services (OR 1.7, 95% CI 1.2 to 2.4); or who terminated the call (OR 1.23; 95% CI 1.2 to 1.3). These three groups were also less likely to be admitted following an ED attendance if they were advised by a CHA (primary care OR 0.89; 95% CI 0.8 to 0.9; other service OR 0.64; 95% CI 0.4 to 0.9; call terminated OR 0.78; 95% CI 0.7 to 0.8; online supplemental figure S4.4). Patients who were advised to attend ED and did so were more likely to be admitted if they were advised by a CHA (OR 1.2; 95% CI 1.17 to 1.3).

Finally, patients who spoke with a CHA were less likely to be otherwise admitted to hospital within 7 days if recommended guardian/self-care (OR 0.7; 95% CI 0.5 to 0.96), primary care (OR 0.68; 95% CI 0.65 to 0.71), other service (OR 0.33; 95% CI 0.2 to 0.5) or if they terminated the call (OR 0.45; 95% CI 0.4 to 0.5; online supplemental figure S4.5).

### Sensitivity analyses

1. The complete case analysis excluded 44 894 calls (4.6%). The resulting adjusted ORs are presented in online supplemental table S5.1. All results indicated differences of similar magnitude and same direction as the main analysis.
2. For callers missing an ED urgency classification, we manually imputed this as described above and reran the analysis. Adjusted ORs for this analysis are in online supplemental table S5.2 and are consistent with findings from the main analysis.
3. Details explaining the final sensitivity analysis are given in online supplemental material S5.3. This sensitivity analysis reclassified the call handler for 2106 calls and excluded 126 (online supplemental figure S5.3). Adjusted ORs for the modified data set are shown in online supplemental table S5.3. Results were consistent with the primary analysis.

## Discussion

This is the first study to examine advisor risk-aversion, triaging accuracy and patient compliance with NHS 111 advice in a single paediatric cohort. Findings suggest that CHAs are markedly less risk-averse in triaging, being less likely to advise people to attend ED or primary care and 45 times as likely to advise guardian/self-care. Patient journeys through ED suggest that CHA triaging is more accurate than NHA triaging: patients who were advised to attend ED and did so were more likely to be admitted if advised by a CHA, indicating CHAs were better at identifying the most urgent cases. Moreover, despite CHAs advising many more patients stay home, those that ignored this advice and attended ED were no less likely to be considered non-urgent and no more likely to be admitted, if advised by a CHA. This indicates that CHAs accurately identified more non-urgent cases. CHAs may also better identify likely deterioration, since other admissions within 7 days were less likely following a low-acuity recommendation given by a CHA. Patients were also more compliant with advice to avoid ED that was given by a CHA.

Findings also suggest that call-terminators, despite not completing the consultation process, are influenced by the advisor type. This group avoided ED more often and was lower acuity if they did attend, if advised by a CHA. It is possible that this cohort consists mainly of the ‘worried well’ who may desire a higher acuity recommendation. If some indication of a low-acuity outcome is apparent during triage, these callers may feel the call is unhelpful and hang up; however, they are still more likely to avoid ED if speaking with a CHA. CHAs may thus be better at identifying the worried well and diverting them from ED via pre-recommendation discussion.

Overall, this study provides evidence that use of CHAs at NHS 111 would be likely to safely reduce paediatric patient demand at ED.

The finding that CHAs typically provide lower-acuity recommendations is consistent with our previous study examining adults, and other research including findings that 74% of calls passed to a CHA for secondary triage were downgraded,^13^ and that general practitioners downgraded advice to attend ED in 73% of cases when reviewing calls.^14^ This evidence, coupled with the finding that patients are more likely to attend ED when advised to stay home by an NHA, supports claims that NHS 111 may be creating unnecessary demand for UEC.^2^

Our finding that patients given low-acuity recommendations by CHAs were less likely to be admitted to hospital within 7 days is novel and suggests that CHAs are better able to predict potential deterioration. Thus, although CHAs recommend guardian/self-care more frequently, this advice may be better targeted than the same advice given by NHAs. Although NHS 111 for paediatric patients is evidenced as safe,^15^ more extensive use of CHAs may improve the balance between patient safety and appropriate UEC use.

Our findings align with an emerging pattern that the more specialised the advisor, the less risk-averse and more accurate their triaging. One study showed emergency medicine consultants were less risk-averse than non-physician CHAs, who in turn were less risk-averse than NHAs.^16^ Another study found that for paediatric NHS 111 calls, paediatric clinicians were less risk-averse than non-paediatric clinicians.^17^ This implies that there is an important cost-benefit balance to be achieved between the specialism of the advisory team and the availability and cost of staffing.

Better patient compliance with CHAs also supports previous research, including findings that patients were less likely to make non-urgent ED attendances following guardian/self-care or primary care recommendations if they received clinical input.^18^ However, high levels of compliance are only desirable if the advice is accurate; if NHA advice is less accurate, patients may be correct to disregard this advice.

Compliance with NHS 111 advice overall may be low: one study found patient compliance of 49% but did not separate by advisor type.^19^ Trust and communication are likely important in maximising compliance. In addition to being potentially distrustful of the Pathways algorithm,^10^ around 25% of parents surveyed did not have confidence in the first advisor they spoke with.^20^ However, when the recommendation was clearly explained, parents were more likely to follow advice. Similarly, communication problems were shown to reduce the likelihood that callers can meaningfully process the information provided by advisors,^21^ with potential implications for compliance. Compliance may therefore be improved both by increased use of CHAs and by improving advisor communication strategies.

### Limitations

This study used data collected before the COVID-19 pandemic. Caller behaviour following an NHS 111 call may have changed since then, so the scope of conclusions may be limited. While we linked ED attendances and acute admissions to NHS 111 calls, we could not examine whether patients/guardians first contacted their primary care provider, so our conclusions may lack nuance. We were unable to determine whether NHAs or the Pathways algorithm is the main source of risk-averse triaging. The definition of non-urgent ED attendances, while well-established, may apply less well to paediatric patients, in that children may benefit from observation in ED without experiencing investigations or treatments. Finally, while our adjustment set accounted for all available important confounders, observational data naturally leads to the possibility of residual confounding.

### Clinical implications

NHS 111 triaging for paediatric patients, and patient compliance, might be significantly improved by greater use of CHAs. This would be likely to maintain or even improve patient safety, while reducing unnecessary ED attendances and ambulance dispatches. This evidence could support workforce planning, help commissioners of integrated urgent care processes in decision-making and help pinpoint strategies to reduce unnecessary UEC use while minimising additional costs. For example, certain calls could be reviewed by CHAs as a matter of course, such as those concerning problems most often resulting in unnecessary ED attendances. Additional communication training for call advisors may also improve patient compliance via enhanced trust and understanding.

### Future research

We plan to replicate this analysis using recent and widespread data to examine whether these trends persist and are generalisable country-wide. We will examine presentation codes in more detail to understand patterns in the origins of unnecessary attendances, and in low-acuity recommendations later recognised as urgent at ED. Further research into terminated calls is required to better understand this cohort and their reasons for termination, and to establish the mechanisms by which they are influenced before a recommendation. Qualitative work could elucidate whether risk aversion originates with NHAs or from constraints within the NHS Pathways framework. A cost-benefit analysis could examine whether reductions in demand for emergency care facilities would justify an investment in more clinical staff to optimise the balance of specialists and service viability.

## Supplementary material

10.1136/archdischild-2025-328896 online supplemental file 1
