# Supplementary material for: Clinical advisors at NHS 111 improve accuracy for paediatric patients and their advice is more reliably followed: a retrospective observational cohort study
Source: Arch Dis Child. 2025 Oct 22;111(2):e328896. doi: 10.1136/archdischild-2025-328896 (PMC12911626; doi:10.1136/archdischild-2025-328896)
Supplement: online supplemental file 1 [file archdischild-111-2-s001.docx]

# Supplementary material

## S1: Categorisation of presenting complaints

**S1 Table: List of symptom group codes and classifications given for regression analysis**

| Symptom descriptor code | Description | Category |
| --- | --- | --- |
| 1206 | NHS Pathways in House Clinician | Other |
| 1207 | Reception Point DO NOT USE | Other |
| 1216 | Other | Other |
| 1218 | Entry pathway | Other |
| 1219 | GP Urgent, Bed Bureau, Interhospital Transfers | Other |
| 1221 | Ambulance Dispatch | Other |
| 1222 | Healthcare Professional Callback | Other |
| 1159 | Symptoms without specific Pathway | Other |
| 1140 | Predetermined Management Plan | Other |
| 1102 | GP Urgent and Bed Bureau | Other |
| 1191 | Health and Social Information | Other |
| 1188 | Deceased | Other |
| 1000 | Abdominal or Flank Injury, Blunt | Injury |
| 1001 | Abdominal or Flank Injury, Blunt, Pregnant | Injury |
| 1002 | Abdominal or Flank Injury, Penetrating | Injury |
| 1003 | Abdominal or Flank Injury, Penetrating, Pregnant | Injury |
| 1011 | Ankle or Foot Injury, Blunt | Injury |
| 1012 | Ankle or Foot Injury, Penetrating | Injury |
| 1013 | Ankle or Foot Pain or Swelling | Injury |
| 1014 | Arm Injury, Blunt | Injury |
| 1015 | Arm Injury, Penetrating | Injury |
| 1020 | Bites or Stings, Insect or Spider | Injury |
| 1021 | Bites, Animal | Injury |
| 1022 | Bites, Human | Injury |
| 1023 | Bites, Snake | Injury |
| 1037 | Burn, Chemical | Injury |
| 1038 | Burn, Thermal | Injury |
| 1040 | Chest or Upper Back Injury, Blunt | Injury |
| 1041 | Chest or Upper Back Injury, Penetrating | Injury |
| 1052 | Dental Injury | Injury |
| 1068 | Eye Injury, Blunt | Injury |
| 1069 | Eye Injury, Penetrating | Injury |
| 1085 | Finger or Thumb Injury, Blunt | Injury |
| 1086 | Finger or Thumb Injury, Penetrating | Injury |
| 1164 | Toe Injury, Blunt | Injury |
| 1165 | Toe Injury, Penetrating | Injury |
| 1183 | Nail Injury | Injury |
| 1087 | Fingernail Injury | Injury |
| 1098 | Genital Injury, Blunt, Pregnant | Injury |
| 1099 | Genital Injury, Penetrating | Injury |
| 1100 | Genital Injury, Penetrating, Pregnant, Over 20 weeks | Injury |
| 1107 | Hand or Wrist Injury, Blunt | Injury |
| 1108 | Hand or Wrist Injury, Penetrating | Injury |
| 1110 | Head, Facial or Neck Injury, Blunt | Injury |
| 1111 | Head, Facial or Neck Injury, Penetrating | Injury |
| 1122 | Lower Back Injury, Blunt | Injury |
| 1123 | Lower Back Injury, Blunt, Pregnant | Injury |
| 1124 | Lower Back Injury, Penetrating | Injury |
| 1145 | Scratches and Grazes | Injury |
| 1211 | Eye splash injury, chemical | Injury |
| 1223 | Bites and Stings | Injury |
| 1097 | Genital Injury, Blunt | Injury |
| 1119 | Leg Injury, Blunt | Injury |
| 1120 | Leg Injury, Penetrating | Injury |
| 1125 | Lower Back Injury, Penetrating, Pregnant | Injury |
| 1071 | Eye Splash Injury or Minor Foreign Body | Injury |
| 1091 | Foreign Body, Ear | Injury |
| 1092 | Foreign Body, Ingested or Inhaled | Injury |
| 1093 | Foreign Body, Nose | Injury |
| 1094 | Foreign Body, Penis | Injury |
| 1095 | Foreign Body, Rectum | Injury |
| 1096 | Foreign Body, Vaginal | Injury |
| 1198 | Stings, Water Creature | Injury |
| 1150 | Skin, Glued | Injury |
| 1066 | Electrical Injury | Injury |
| 1132 | Nosebleeds, Traumatic | Injury |
| 1167 | Toenail Injury | Injury |
| 1200 | Frequent Caller | Mixed |
| 1042 | Child Safeguard/Protection or Vulnerable Adult Concern | Mixed |
| 1172 | Unwell, Under 1 Year Old | Mixed |
| 1186 | Worsening known Mental health problem | Mixed |
| 1084 | Fever | Mixed |
| 1088 | Fits Within the Last 12 Hours | Mixed |
| 1112 | Headache | Mixed |
| 1113 | Headache, Pregnant | Mixed |
| 1170 | Toxic Ingestion/Inhalation/Overdose | Mixed |
| 1004 | Abdominal Pain | Mixed |
| 1005 | Abdominal Pain, Pregnant, Over 20 Weeks | Mixed |
| 1006 | Abdominal, Flank, Groin or Back Pain or Swelling | Mixed |
| 1010 | Allergic Reaction | Mixed |
| 1034 | Breathing Problems, Breathlessness or Wheeze | Mixed |
| 1035 | Breathing Problems, Breathlessness or Wheeze, Pregnant | Mixed |
| 1101 | Genital Problems | Mixed |
| 1117 | Knee or Lower Leg Pain or Swelling | Mixed |
| 1118 | Labour and Childbirth | Mixed |
| 1137 | Palpitations | Mixed |
| 1138 | Palpitations, Pregnant | Mixed |
| 1152 | Skin, Rash | Mixed |
| 1175 | Vaginal Bleeding, Pregnant | Mixed |
| 1184 | Abdominal Pain, Rectal Bleeding, Pregnant Over 20 Weeks | Mixed |
| 1144 | Rectal Pain, Swelling, Lump or Itch | Mixed |
| 1196 | Declared Seizure Warning | Mixed |
| 1203 | Mental Health Problem | Mixed |
| 1226 | Dental Problems | Mixed |
| 1230 | Cold Exposure | Mixed |
| 1025 | Blood in Urine | Mixed |
| 1128 | Lower Limb Pain or Swelling | Mixed |
| 1054 | Diarrhoea | Mixed |
| 1055 | Diarrhoea and Vomiting | Mixed |
| 1056 | Diarrhoea or Vomiting, Pregnant, Over 20 Weeks | Mixed |
| 1126 | Lower Back Pain | Mixed |
| 1127 | Lower Back Pain, Pregnant, Over 20 weeks | Mixed |
| 1143 | Rectal Bleeding | Mixed |
| 1009 | Alcohol Intoxication | Mixed |
| 1059 | Dizziness or Vertigo | Mixed |
| 1060 | Dizziness or Vertigo Pregnant | Mixed |
| 1179 | Vomiting | Mixed |
| 1187 | Diabetes, Blood Sugar Problem | Mixed |
| 1047 | Cough | Mixed |
| 1048 | Cough, Pregnant | Mixed |
| 1050 | Deliberate Self-Harm | Mixed |
| 1115 | Hip, Thigh or Buttock Pain or Swelling | Mixed |
| 1147 | Shoulder Pain | Mixed |
| 1053 | Diabetic Hypoglycaemia | Mixed |
| 1089 | Flank or Side Pain | Mixed |
| 1090 | Flank or Side Pain, Pregnant | Mixed |
| 1129 | Mouth Ulcers | Mixed |
| 1133 | Numbness or Pins and Needles | Mixed |
| 1018 | Behaviour Change | Mixed |
| 1019 | Behaviour or Mood disturbance, Pregnant | Mixed |
| 1057 | Difficulty Passing Urine | Mixed |
| 1058 | Difficulty Swallowing | Mixed |
| 1065 | Easy or Unexplained Bruising | Mixed |
| 1067 | External Fixation Problems | Mixed |
| 1079 | Face and Neck Pain or Swelling | Mixed |
| 1080 | Face, Neck Pain or Swelling | Mixed |
| 1081 | Falls or Faints Without Injury | Mixed |
| 1082 | Falls or Faints Without Injury, Pregnant | Mixed |
| 1103 | Groin Pain or Groin Swelling | Mixed |
| 1104 | Groin Pain or Groin Swelling, Pregnant, Under 20 Weeks | Mixed |
| 1105 | Groin Pain or Swelling, Pregnant, Over 20 Weeks | Mixed |
| 1142 | Rash | Mixed |
| 1157 | Stoma Problems | Mixed |
| 1171 | Tube and Drain Problems | Mixed |
| 1173 | Urinary catheter Problems | Mixed |
| 1174 | Vaginal Bleeding | Mixed |
| 1185 | Drug, solvent, alcohol misuse | Mixed |
| 1210 | Tremor | Mixed |
| 1227 | Wound Problems, Plaster Casts, Tubes and Metal Appliances | Mixed |
| 1228 | Self-Harm | Mixed |
| 1192 | Repeat Prescription | Non-urgent |
| 1155 | Social or Domestic Emergency | Non-urgent |
| 1131 | Nosebleeds without injury | Non-urgent |
| 1139 | Plaster Cast Problems | Non-urgent |
| 1156 | Sore Throat and Hoarse Voice | Non-urgent |
| 1213 | Hiccups | Non-urgent |
| 1106 | Hair loss | Non-urgent |
| 1109 | Head Lice | Non-urgent |
| 1024 | Blisters | Non-urgent |
| 1130 | Nasal Congestion | Non-urgent |
| 1151 | Skin, Minor Foreign Body | Non-urgent |
| 1154 | Sleep Difficulties | Non-urgent |
| 1161 | Teething | Non-urgent |
| 1205 | Hearing Problems or Blocked Ear | Non-urgent |
| 1208 | Pubic Lice | Non-urgent |
| 1209 | Scabies | Non-urgent |
| 1008 | Acne, Spots and Pimples | Non-urgent |
| 1017 | Athlete's Foot | Non-urgent |
| 1075 | Eye, Sticky, Watery | Non-urgent |
| 1078 | Eyelid Problems | Non-urgent |
| 1134 | Other Dental Problems - Fillings, Crowns Bridges, Appliances etc | Non-urgent |
| 1146 | Sexual Problems or Concerns | Non-urgent |
| 1148 | Sinusitis | Non-urgent |
| 1149 | Skin Lumps | Non-urgent |
| 1160 | Tattoos, Birthmarks or Moles | Non-urgent |
| 1074 | Eye, Sticky or Watery | Non-urgent |
| 1162 | Tiredness (Fatigue) | Non-urgent |
| 1163 | Tiredness (Fatigue), Pregnant | Non-urgent |
| 1176 | Vaginal Discharge | Non-urgent |
| 1178 | Vaginal Swelling | Non-urgent |
| 1026 | Breast Discharge | Non-urgent |
| 1027 | Breast Discharge, Pregnant | Non-urgent |
| 1028 | Breast Lump | Non-urgent |
| 1029 | Breast Lump, Pregnant | Non-urgent |
| 1032 | Breast Problems | Non-urgent |
| 1045 | Constipation | Non-urgent |
| 1046 | Constipation, Pregnant | Non-urgent |
| 1177 | Vaginal Itch or Soreness | Non-urgent |
| 1182 | Wrist, Hand or Finger Pain or Swelling | Non-urgent |
| 1061 | Drowsiness | Non-urgent |
| 1062 | Drowsiness, Pregnant | Non-urgent |
| 1007 | Absent or Missed Period | Non-urgent |
| 1197 | Faecal Incontinence | Non-urgent |
| 1158 | Sunburn | Non-urgent |
| 1166 | Toe Pain or Swelling | Non-urgent |
| 1030 | Breast Pain | Non-urgent |
| 1031 | Breast Pain, Pregnant | Non-urgent |
| 1064 | Earache | Non-urgent |
| 1063 | Ear Discharge or Ear Wax | Non-urgent |
| 1070 | Eye or Eyelid Problems | Non-urgent |
| 1043 | Cold or Flu | Non-urgent |
| 1044 | Cold or Flu, Pregnant | Non-urgent |
| 1116 | Itch | Non-urgent |
| 1135 | Pain and/or Frequency Passing Urine | Non-urgent |
| 1136 | Pain, Frequency and/or Difficulty Passing Urine | Non-urgent |
| 1168 | Toothache After Dental Injury | Non-urgent |
| 1169 | Toothache Without Dental Injury | Non-urgent |
| 1224 | Eye or Eyelid Problems | Non-urgent |
| 1229 | Dental Module | Non-urgent |
| 1204 | Failed Contraception | Non-urgent |
| 1225 | Skin Problems | Non-urgent |
| 1033 | Breastfeeding Problems | Non-urgent |
| 1051 | Dental Bleeding | Non-urgent |
| 1083 | Falls Without Injury | Non-urgent |
| 1114 | Heat Exposure | Non-urgent |
| 1121 | Locked Jaw | Non-urgent |
| 1072 | Eye, Painful | Non-urgent |
| 1073 | Eye, Red or Irritable | Non-urgent |
| 1016 | Arm, Pain or Swelling | Non-urgent |
| 1181 | Wound Problems | Non-urgent |
| 1180 | Vomiting Blood | Urgent |
| 1190 | Major Trauma | Urgent |
| 1193 | Immediate threats to life | Urgent |
| 1194 | Trauma Emergency | Urgent |
| 1212 | Implantable Cardioverter Defibrillator Shock | Urgent |
| 1217 | Stroke Like Symptoms | Urgent |
| 1049 | Coughing up Blood | Urgent |
| 1231 | Drowning Incident | Urgent |
| 1036 | Bringing Up Blood | Urgent |
| 1141 | Probable Stroke | Urgent |
| 1202 | Limb, cold or colour change | Urgent |
| 1076 | Eye, Visual Loss or Disturbance | Urgent |
| 1077 | Eye, Yellow | Urgent |
| 1153 | Skin, Yellow | Urgent |
| 1189 | Dying | Urgent |
| 1201 | Reduced Fetal Movements | Urgent |
| 1195 | Non-trauma Emergency | Urgent |
| 1039 | Chest and Upper Back Pain | Urgent |

## S2: Demographics breakdown

**S2 Table. Demographics and outcomes 2-5 split by recommendation given.** Note percentages in this table are row-wise to illustrate recommendation split between each demographic/outcome category.

|  | Guardian/  Self-care | Primary Care | Attend  ED | Ambulance dispatch | Other | Call  terminated |
| --- | --- | --- | --- | --- | --- | --- |
|  | N=87,169 | N=632,930 | N=90,552 | N=44,452 | N=7,510 | N=109,608 |
| Age (Median, IQR) | 2 [1, 2] | 2 [1, 2] | 3 [1, 3] | 2 [0, 2] | 3 [0, 3] | 1 [0, 1] |
| Gender (N, %) |  |  |  |  |  |  |
| Male | 45,628 (9.0) | 326,634 (64) | 49,261 (9.3) | 24,551 (4.8) | 3,921 (0.8) | 58,681 (11) |
| Female | 41,511 (9.0) | 306,255 (67) | 41,288 (9.7) | 19,897 (4.3) | 3,583 (0.8) | 46,834 (11) |
| Not stated/  Indeterminate | 30 (0.7) | 41 (1.0) | 3 (0.1) | 4 (0.1) | 6 (0.1) | 4,093 (98) |
| IMD Quintile (N, %) |  |  |  |  |  |  |
| 1 (Most deprived) | 31,780 (8.4) | 256,669 (68) | 35,004 (9.3) | 19,841 (5.3) | 3,040 (0.8) | 31,449 (8.3) |
| 2 | 17,034 (9.2) | 124,234 (67) | 17,698 (9.6) | 8,793 (4.8) | 1,437 (0.8) | 15,466 (8.4) |
| 3 | 13,823 (9.8) | 93,646 (66) | 14,076 (10) | 6,173 (4.4) | 1,140 (0.8) | 12,077 (8.6) |
| 4 | 13,823 (10) | 92,025 (67) | 13,635 (9.9) | 5,677 (4.1) | 1,109 (0.8) | 11,550 (8.4) |
| 5 (Least deprived) | 10,167 (10) | 64,629 (66) | 9,890 (10) | 3,848 (4) | 743 (0.8) | 8,131 (8.3) |
| None recorded | 542 (1.6) | 1,727 (5.1) | 249 (0.7) | 120 (0.4) | 41 (0.1) | 30935 (92) |
| Complaint category (N, %) | |  |  |  |  |  |
| Non-urgent | 18,164 (7.9) | 197,402 (85) | 4,944 (2.1) | 609 (0.3) | 41 (<0.1) | 9,986 (4.3) |
| Urgent | 475 (1.1) | 12,635 (30) | 5,157 (12) | 1,7341 (40) | 61 (0.1) | 7,216 (17) |
| Injury | 10,872 (12.2) | 11,573 (13) | 52,330 (59) | 4,589 (5.1) | 4 (<0.1) | 9,829 (11) |
| Mixed/unclear | 50,431 (9.4) | 400,938 (75) | 25,567 (4.8) | 21,612 (4.0) | 247 (<0.1) | 36,919 (6.9) |
| Other | 7,180 (18) | 10,272 (26) | 2,523 (6.3) | 263 (0.7) | 7,156 (18) | 12,820 (32) |
| None recorded | 47 (0.1) | 110 (0.3) | 31 (0.1) | 38 (0.1) | 1 (<0.1) | 32,838 (99) |
| Time of call (N, %) |  |  |  |  |  |  |
| In-hours | 22,889 (11) | 114,223 (56) | 25,312 (13) | 8,582 (4.2) | 597 (0.3) | 31,151 (15) |
| Out-of-hours | 64,280 (8.4) | 518,707 (67) | 65,240 (8.5) | 35,870 (4.7) | 6,913 (0.9) | 78,457 (10) |
| Attended ED within 48h | 2,325 (1.2) | 49,828 (26) | 71,492 (37) | 36,373 (19) | 880 (0.5) | 31,432 (16) |
| ED urgency (N, %) |  |  |  |  |  |  |
| Non-urgent | 656 (1.5) | 10,944 (24) | 19,862 (44) | 6,406 (14) | 212 (0.5) | 7,105 (16) |
| Urgent | 1,415 (1.1) | 33,521 (27) | 43,313 (34) | 26,668 (21) | 577 (0.5) | 20,981 (17) |
| Not calculable | 254 (1.2) | 5,363 (26) | 8,317 (40) | 3,299 (16) | 91 (0.4) | 3,346 (16) |
| Admitted from ED  (N, %) | 367 (0.9) | 13,794 (33) | 9,071 (21) | 11,854 (28) | 228 (0.5) | 6,890 (16) |
| Otherwise admitted in 7d (N, %) | 514 (1.8) | 22,660 (80) | 1,688 (5.9) | 1,657 (5.8) | 187 (0.7) | 1,730 (6.1) |

## S3: Unadjusted results

**S3 Table: Unadjusted odds ratios, 95% confidence intervals and N included for each regression.** Reference level was NHA. *Caveats as described in Table 2 in main text.

|  | Guardian/  self-care | Primary  Care | Attend  ED | Ambulance dispatch | Other | Call  terminated |
| --- | --- | --- | --- | --- | --- | --- |
| Received recommendation | 47  (46, 49) | 0.307  (0.304, 0.31) | 0.86  (0.84,0.87) | 0.44  (0.43, 0.45) | 2.2  (2.1, 2.3) | 1.1  (1.09, 1.12) |
| Attended ED within 48 hours | 0.69  (0.6, 0.8)  N = 87,169 | 0.79  (0.77, 0.81)  N = 632,930 | 1.12  (1.08, 1.16)  N = 90,552 | 0.85  (0.80, 0.91)  N = 44,452 | 0.74  (0.64, 0.86)  N = 7,510 | 0.44  (0.42, 0.45)  N = 109,608 |
| Classified as  non-urgent* | 0.9  (0.7, 1.2)  N = 2,071 | 1.09  (1.03, 1.16)  N = 44,465 | 0.89  (0.86, 0.93)  N = 63,175 | 1.03  (0.95, 1.11)  N = 33,074 | 2.2  (1.6, 3)  N = 789 | 1.3  (1.2, 1.4)  N = 28,086 |
| Admitted  from ED | 1.2  (0.8, 1.7)  N = 2,325 | 0.87  (0.82, 0.92)  N = 49,828 | 2.1  (2.0, 2.2)  N = 71,492 | 0.9  (0.84, 0.96)  N = 36,373 | 0.62  (0.45, 0.85)  N = 880 | 0.79  (0.73, 0.85)  N = 31,432 |
| Otherwise admitted in 7d | 0.48  (0.44, 0.52)  N = 196,777 | 0.68  (0.65, 0.71)  N = 632,930 | 1.4  (1.2, 1.5)  N = 90,552 | 1  (0.9, 1.1)  N = 44,452 | 0.3  (0.2, 0.5)  N = 7,510 | 0.8  (0.7, 0.9)  109,608 |

## S4: Additional figures


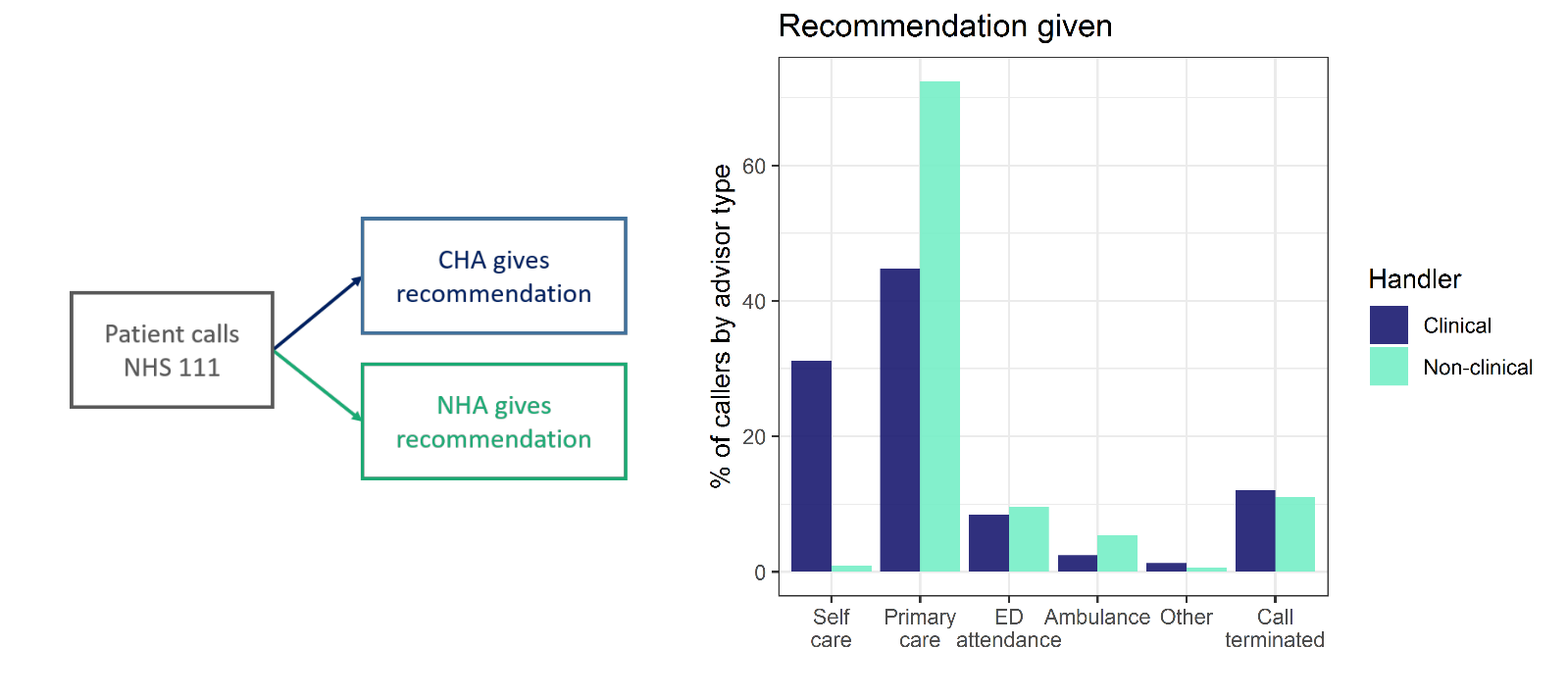


**S4.1 fig: Percentage of callers receiving each recommendation according to advisor type.**


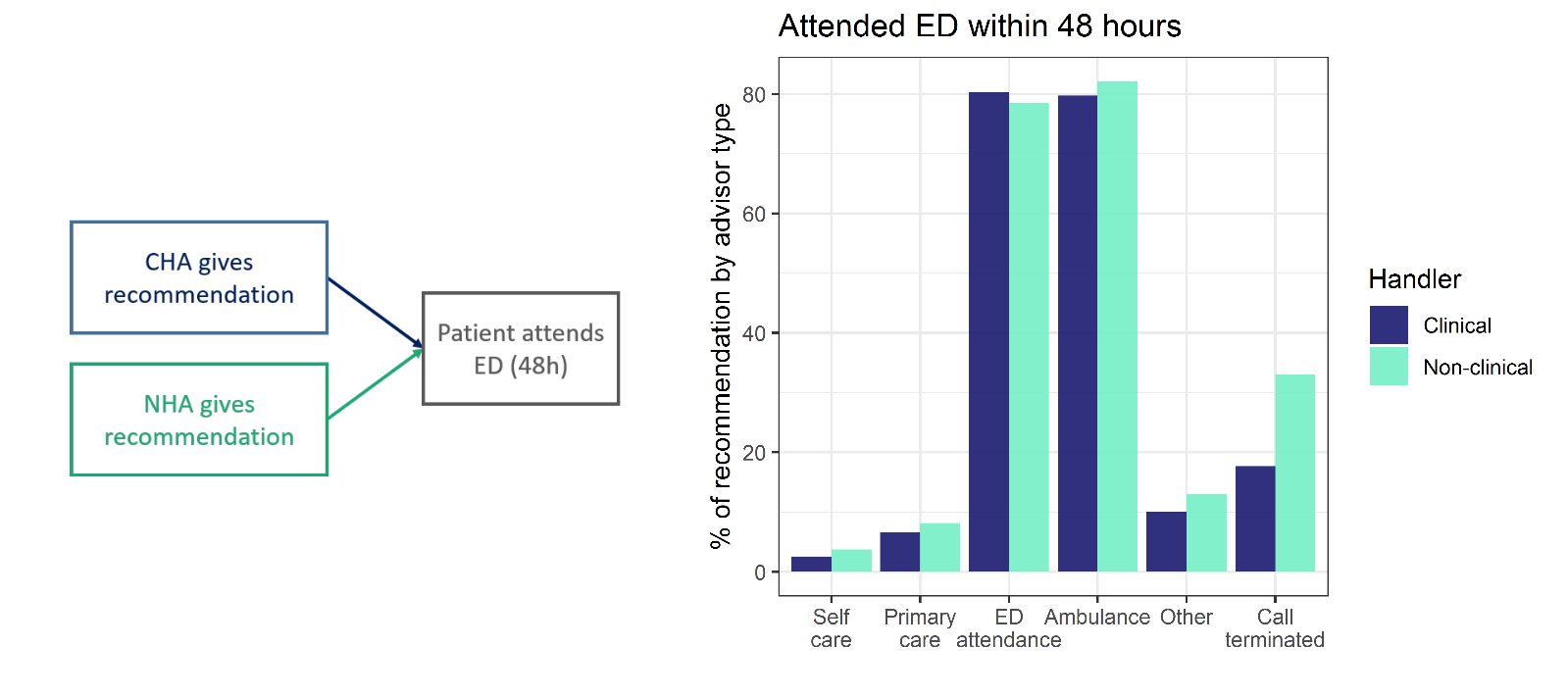


**S4.2 fig: Percentage of callers attending ED by recommendation and advisor type.** Numerator: N ED attendances from recommendation*advisor type group; Denominator: total N of recommendation*advisor type group.


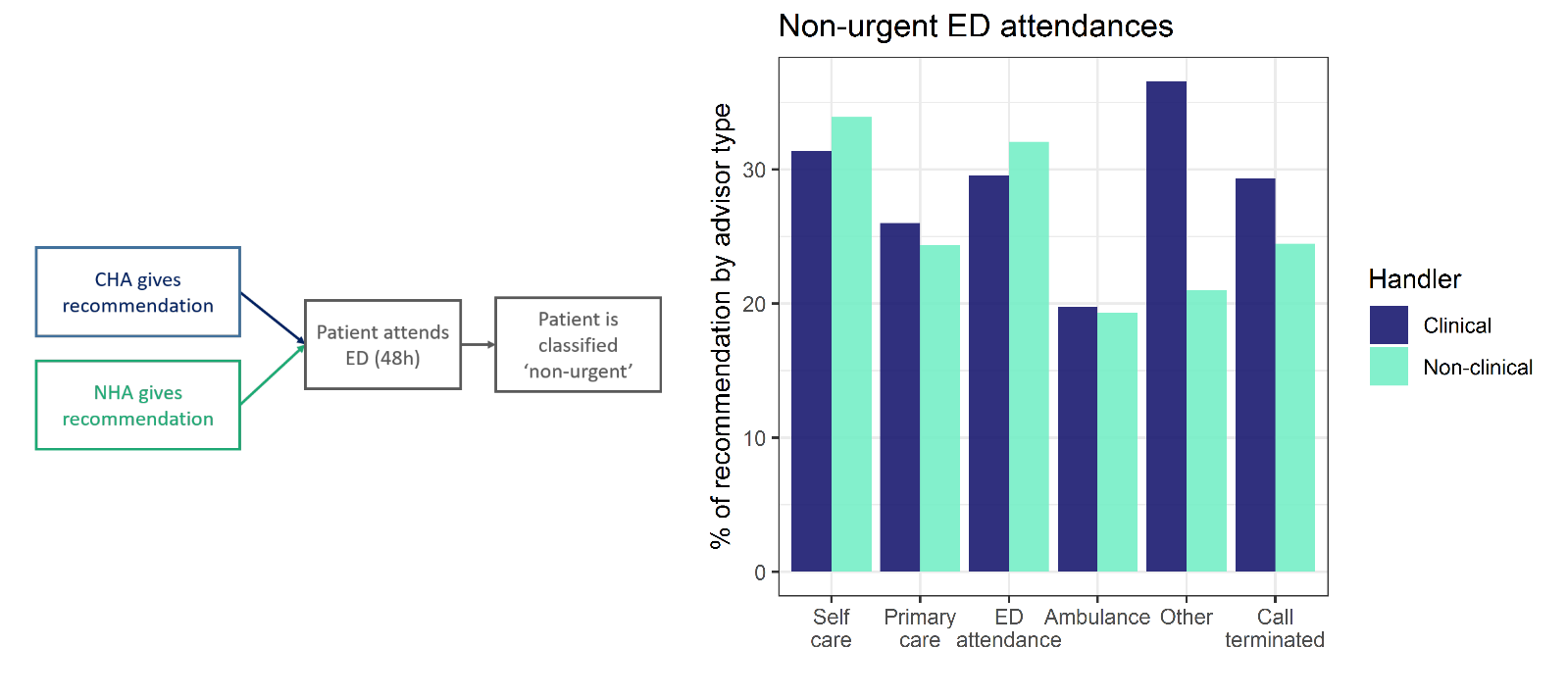
**S4.3 fig: Percentage of callers receiving a non-urgent classification after attending ED, by recommendation and advisor type.** Numerator: N non-urgent ED attendances from recommendation*advisor type group; Denominator: N ED attendances from recommendation*advisor type group.


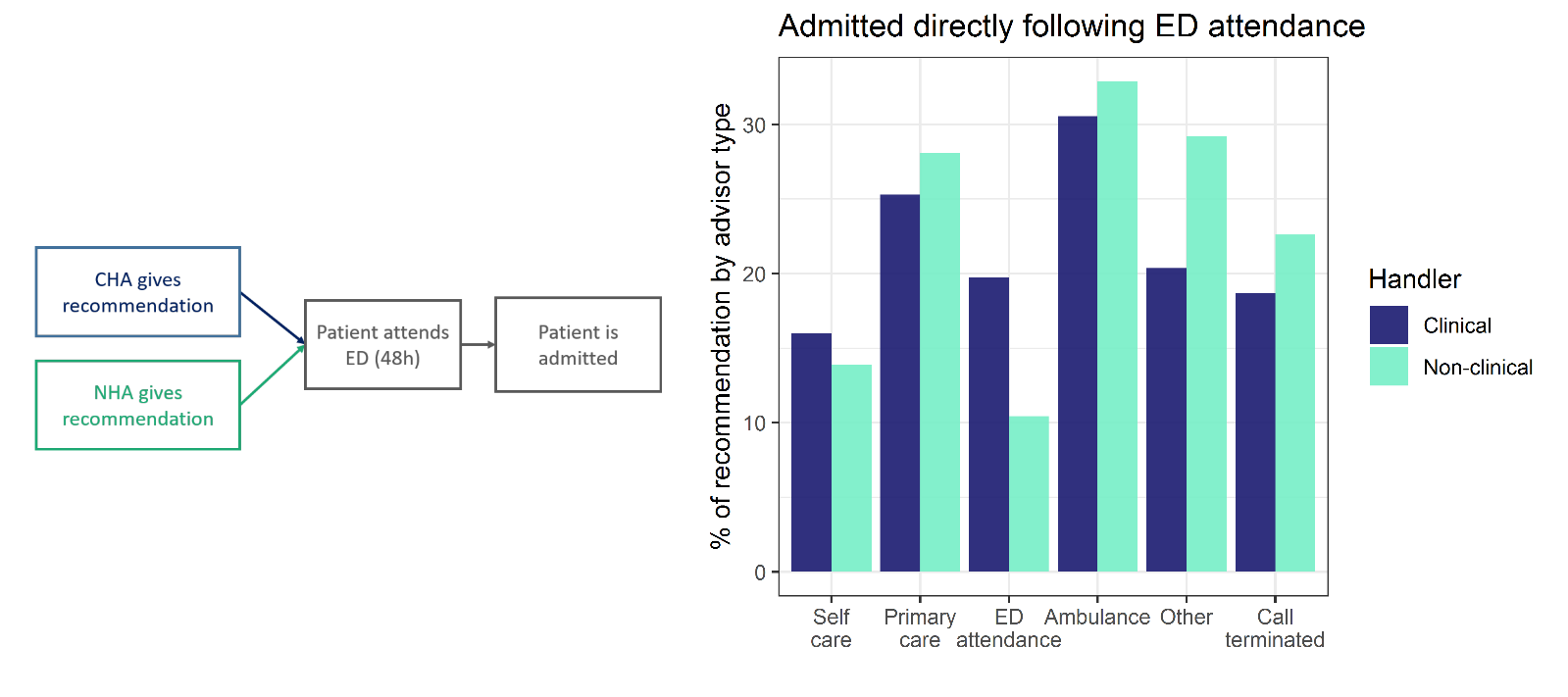


**S4.4 fig: Percentage of callers admitted to hospital after attending ED, by recommendation and advisor type**. Numerator: N callers admitted after an ED attendance, from recommendation*advisor type group; Denominator: N ED attendances from recommendation*advisor type group.


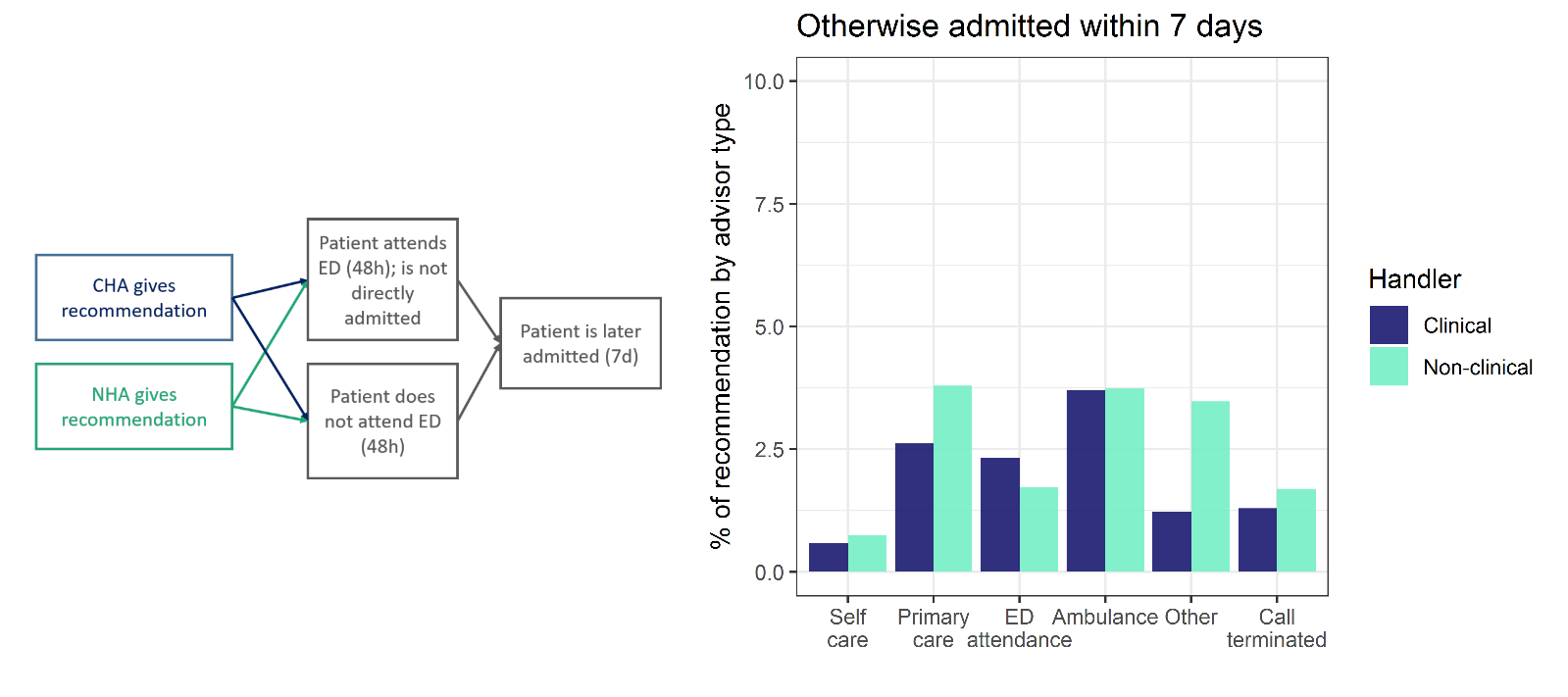


**S4.5 fig: Percentage of callers admitted indirectly to hospital within 7 days, by recommendation and advisor type.** Numerator: N callers admitted within 7 days, but not directly from an ED attendance within 48 hours, from recommendation*advisor type group; Denominator: total N of recommendation*advisor type group.

## S5: Sensitivity analyses

**S5.1 Table: odds ratios, 95% confidence intervals and N included for sensitivity analysis i: complete case analysis.**

|  | Guardian/  self-care | Primary  Care | Attend  ED | Ambulance dispatch | Other | Call  terminated |
| --- | --- | --- | --- | --- | --- | --- |
| Received recommendation  N = 927,327 | 45  (44, 46) | 0.161  (0.159, 0.163) | 0.78  (0.76, 0.79) | 0.5  (0.48, 0.51) | 0.76  (0.72, 0.8) | 2.18  (2.15, 2.22) |
| Attended ED within 48 hours | 0.63  (0.55, 0.73)  N = 86,573 | 0.78  (0.76, 0.8)  N = 631,056 | 1  (0.96, 1.05)  N = 90,269 | 0.9  (0.85, 1)  N = 44,299 | 0.8  (0.7, 0.9)  N = 7,465 | 0.123  (0.12, 0.13)  N = 67,665 |
| Classified as  non-urgent* | 0.9  (0.7, 1.2)  N = 2,065 | 1.07  (1.0, 1.1)  N = 44,376 | 0.97  (0.93, 1.02)  N = 63,035 | 1  (0.9, 1.1)  N = 33,012 | 1.7  (1.2, 2.4)  N = 788 | 1.23  (1.1, 1.3)  N = 26,801 |
| Admitted  from ED | 1.2  (0.8, 1.8)  N = 2,318 | 0.89  (0.8, 0.9)  N = 49,727 | 1.2  (1.17, 1.3)  N = 71,337 | 0.95  (0.9, 1.0)  N = 36,306 | 0.63  (0.4, 0.9)  N = 878 | 0.78  (0.7, 0.8)  N = 29,973 |
| Otherwise admitted in 7d | 0.7  (0.5, 1.0)  N = 86,573 | 0.68  (0.66, 0.71)  N = 631,056 | 0.94  (0.8, 1.1)  N = 90,269 | 1.0  (0.9, 1.2)  N = 44,299 | 0.33  (0.2, 0.5)  N = 7,465 | 0.44  (0.4, 0.5)  N = 67,665 |

**S5.2 Table: 95% confidence intervals and N included for sensitivity analysis ii: Imputed ED attendance urgency.**

|  | Guardian/  self-care | Primary  Care | Attend  ED | Ambulance dispatch | Other | Call  terminated |
| --- | --- | --- | --- | --- | --- | --- |
| Classified as  non-urgent | 0.9  (0.7, 1.2)  N = 2,325 | 1.09  (1.03, 1.15)  N = 49,828 | 0.96  (0.93, 1.0)  N = 71,492 | 1.0  (0.9, 1.1)  N = 36,373 | 1.4  (1.0, 1.9)  N = 880 | 1.2  (1.1, 1.3)  N = 31,432 |

### **S5.3 Sensitivity analysis iii**

Whether an NHS 111 call was subject to input from a CHA was indicated by a binary flag in the data. However, in addition to this, other fields contained indicators as to whether a CHA handled the call.

These indicators were that:

1. The triage outcome is overridden (indicated by a categorical flag). This action should not be available to NHAs (except when an initial triage results in a recommendation to speak with a CHA, but none is available)
2. The final recommendation is not consistent with the triaging outcome. This should only happen when a triage over-ride has occurred, and again, this action should not be accessible to NHAs.
3. There is a record of the call having been transferred to a CHA or that a CHA called the patient back.

Where one or more of these indicators was present in the data, but the record showed that an NHA handled the call, these were deemed ambiguous calls. In some cases, it was considered likely that a CHA advised the call. These were reclassified as CHA calls for this sensitivity analysis. In other cases, the record was deemed sufficiently ambiguous that it was unclear whether a CHA was involved. These calls were excluded from this sensitivity analysis. S6 fig details the precise circumstances of the reclassifications. Final classifications for this analysis are shown in blue (CHA), green (NHA) and red (excluded) boxes. Reclassified calls are shown with a grey background. Calls with a white background retained the same classification as for the main analysis.

## **
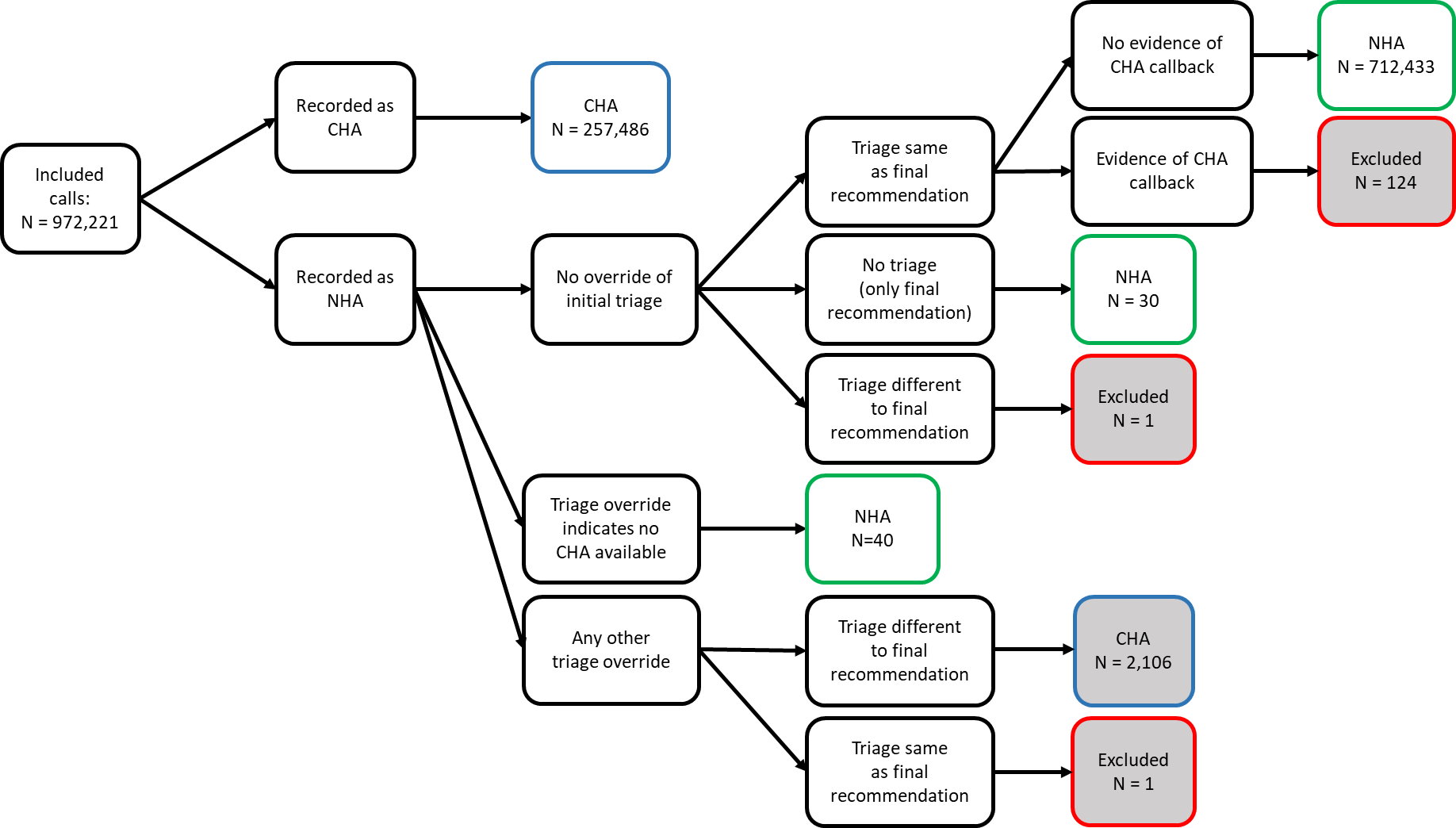
**

**S5.3 fig: Reclassification of calls for sensitivity analysis, for calls recorded as being handled by an NHA**

**S5.3 Table: Odds ratios, 95% confidence intervals and N included for each regression using the reclassified calls dataset.** Reference level was NHA. *,^✝^Caveats as described in Table 2 in main text.

|  | Guardian/  Self-care | Primary  Care | Attend  ED | Ambulance dispatch | Other | Call  terminated |
| --- | --- | --- | --- | --- | --- | --- |
| Received recommendation  N = 972,095 | 44  (43,46) | 0.167  (0.165, 0.17) | 0.78  (0.77, 0.8) | 0.49  (0.48, 0.5) | 0.77  (0.73, 0.81) | 2.0  (1.97, 2.03) |
| Attended ED within 48 hours | 0.64  (0.56, 0.73)  N = 87,166 | 0.8  (0.78, 0.83)  N = 632,860 | 1.0  (0.96, 1.05)  N = 90,548 | 0.91  (0.85, 0.98)  N = 44,450 | 0.79  (0.68, 0.92)  N = 7,505 | 0.128  (0.12, 0.13)  N = 109,566 |
| Classified as  non-urgent* | 0.9  (0.7, 1.2)  N = 2,071 | 1.05  (0.99, 1.12)  N = 44,460 | 0.97  (0.93, 1.02)  N = 63,173 | 1.0  (0.93, 1.1)  N = 33,072 | 1.7  (1.2, 2.4)  N = 789 | 1.2  (1.1, 1.3)  N = 28,082 |
| Admitted  from ED | 1.2  (0.8, 1.8)  N = 2,325 | 0.9  (0.85, 0.95)  N = 49,822 | 1.24  (1.17, 1.3)  N = 71,489 | 0.95  (0.89, 1.02)  N = 36,371 | 0.63  (0.45, 0.89)  N = 880 | 0.78  (0.72, 0.84)  N = 31,427 |
| Otherwise admitted in 7d^✝^ | 0.7  (0.5, 1.0)  N = 87,166 | 0.7  (0.67, 0.73)  N = 632,860 | 0.94  (0.83, 1.1)  N = 90,548 | 1.0  (0.9, 1.2)  N = 44,450 | 0.33  (0.2, 0.5)  N = 7,505 | 0.45  (0.4, 0.5)  109,566 |
